# Feasibility of dried blood spots for HIV viral load monitoring in decentralized area in North Vietnam in a test-and-treat era, the MOVIDA project

**DOI:** 10.1371/journal.pone.0230968

**Published:** 2020-04-09

**Authors:** Tuan Anh Nguyen, Tram Hong Tran, Binh Thanh Nguyen, Tram Thi Phuong Pham, Nhung Thi Hong Le, Dung Viet Ta, Huong Thi Thu Phan, Long Hoang Nguyen, Mohand Ait-Ahmed, Hien Thi Ho, Fabien Taieb, Yoann Madec, for the MOVIDA 2 study group

**Affiliations:** 1 National Reference Laboratory of HIV Molecular Biology, National Institute of Hygiene and Epidemiology, Hanoi, Vietnam; 2 Hanoi University of Public Health, Hanoi, Vietnam; 3 Vietnam Administration of HIV/AIDS Control, Ministry of health, Hanoi, Vietnam; 4 Center for Translational Research, Institut Pasteur, Paris, France; 5 Emerging Diseases Epidemiology Unit, Institut Pasteur, Paris, France; National Institute for Communicable Disease (NICD), South Africa, SOUTH AFRICA

## Abstract

**Background:**

Access to HIV viral load is crucial to efficiently monitor patients on antiretroviral treatment (ART) and prevent HIV drug resistance acquisition. However, in some remote settings, access to viral load monitoring is still complex due to logistical and financial constraints. Use of dried blood spots (DBS) for blood collection could overcome these difficulties. This study aims to describe feasibility and operability of DBS use for routine viral load monitoring.

**Methods:**

From June 2017 to April 2018, HIV-infected adults who initiated ART were enrolled in a prospective cohort in 43 clinical sites across 6 provinces in North Vietnam. Following national guidelines, the first viral load monitoring was planned 6 months after ART initiation. DBS were collected at the clinical site and sent by post to a central laboratory in Hanoi for viral load measurement.

**Results:**

Of the 578 patients enrolled, 537 were still followed 6 months after ART initiation, of which DBS was collected for 397 (73.9%). The median (inter quartile range) delay between DBS collection at site level and reception at the central laboratory was 8 (6–19) days and for 70.0% viral load was measured ≤30 days after blood collection. The proportion of patients with viral load ≥1000 copies/mL at the 6 month evaluation was 15.9% (n = 59). Of these, a DBS was collected again to confirm virological failure in 15 (24.4%) of which virological failure was confirmed in 11 (73.3%).

**Conclusion:**

Delay of DBS transfer to the central laboratory was acceptable and most viral loads were measured in ≤30 days, in-line with routine follow-up. However, the level of DBS coverage and the proportion of patients in failure for whom a confirmatory viral load was available were suboptimal, indicating that integration of viral load monitoring in the field requires, among other things, careful training and strong involvement of the local teams. The proportion of patients experiencing virological failure was in line with other reports; interestingly those who reported being non-adherent and those with a low BMI were more at risk of failure.

## Introduction

In Vietnam, according to the latest UNAIDS report, HIV prevalence has been estimated at 0.3% in the adult population [1]. The epidemic, however, is essentially concentrated in people who inject drug (PWID), men who have sex with men (MSM) and female sex-workers (FSW).

Access to antiretroviral therapy (ART) began in the mid-1990s, and the number of HIV-infected patients on ART rapidly increased in the 2000s. Between 2017 and 2018, it was estimated that the proportion of people infected with HIV who were on ART increased from 50% to 65% [1, 2]. National guidelines, in accordance with the World Health Organization (WHO) guidelines, recommend initiating an ART combination based on two nucleotide reverse transcriptase inhibitors associated with one non-nucleotide reverse transcriptase inhibitor [3].

In 2014, UNAIDS launched the 90-90-90 goals to help end the AIDS epidemic [4]. To increase the number of patients on ART and reach the first two 90 goals, Vietnam initiated a test-and-treat strategy through the implementation of large-scale testing programs and access to ART to all HIV-infected patients, whatever their clinical condition or immuno-virological level.

To help reach the last 90, expanded access to viral load monitoring is crucial. In 2017, it was estimated that about one third of those on ART received viral load testing in Vietnam [5]. This is particularly important as the immunological and clinical criteria to diagnose therapeutic failure have been shown to perform poorly [6–8]. Laboratories currently able to perform viral load measurements are concentrated in the large urban centers of Hanoi and Ho Chi Minh City, while HIV patients are spread all over the country. The gold standard for viral load monitoring relies on plasma samples, but this requires maintaining a cold-chain from the blood sampling site to the laboratory where the viral load will be measured, which are distant in both space and time. This is costly and subject to technical and logistical difficulties. Prior to this study, routine viral load monitoring was not available in remote settings, neither by plasma nor by DBS. To overcome the challenges of plasma transfer, dried blood spots (DBS) which are easy to collect and easy to transfer, have also performed well at detecting virological failure when compared to plasma [9–12].

Hence, the MOVIDA 2 project (**M**onitoring **O**f **V**iral load **I**n **D**ecentralised **A**rea, Clinical trials ID: NCT03249493) was implemented to evaluate, in real-life conditions, the feasibility of DBS use for HIV viral load monitoring in remote provinces in North Vietnam, where no routine HIV viral load monitoring was available. Prior to this project, two laboratory evaluations were conducted establishing that, at the central laboratory in charge of viral load measurement, viral load results on DBS and plasma compared well and fulfilled WHO requirements [13, 14].

In the present study we aimed to describe i) the cohort of patients who initiated ART at the time of test-and-treat in remote areas in Vietnam, ii) the process of DBS transfer from remote areas to the central laboratory and iii) virological outcomes at 6 months of ART.

## Methods

The MOVIDA 2 project, a prospective observational cohort study implemented in 6 provinces in North Vietnam, enrolled HIV infected adults who initiated ART in 43 clinical sites between June 2017 and April 2018. The choice of the provinces and the clinical sites was at the discretion of the Vietnam Administration of HIV/AIDS control (VAAC) which is a structure within the Ministry of Health in Vietnam. Follow-up in the cohort started at the time of ART initiation.

Following national and WHO guidelines, virological monitoring for patients on ART was scheduled at 6 months, 12 months and every 12 months thereafter [3]. Virological failure was defined at the threshold of 1000 copies/mL. In the case of a viral load above that threshold, guidelines are to offer adherence support to the patient and re-control the viral load within 3 to 4 months.

For viral load monitoring, a venous blood puncture was performed at the clinical site; then, using a calibrated pipette, 70μL of whole blood was deposited on each of the 5 pre-perforated spots of two Munktell TFN (Ahlstrom-Munksjö, Bärenstein, Germany) paper cards. The DBS cards were then left to dry for a minimum of 3 hours at ambient temperature before being packed into individual ziplock bags with 3 desiccants. They were then kept at ambient temperature until transfer.

Once a week, all the individual DBS envelopes available at the laboratory were collected into a larger envelope, with the address of the central laboratory pre-printed on it, along with a transfer form on which the name of the clinical site, the date of transfer and the number of DBS sent were indicated. A member of laboratory staff then personally transported this envelope to the nearest post office for postage. DBS cards were thus transferred weekly by postal service to the National Reference laboratory of HIV molecular biology at the National Institute of Hygiene and Epidemiology (NIHE), Hanoi (Vietnam). However, some clinical sites initially transferred the DBS to an intermediate laboratory in the provincial capital which then transferred the DBS to the laboratory in Hanoi. This concerned a limited number of DBS, and this procedure soon stopped after informing the clinical sites to transfer directly to the laboratory in Hanoi Upon DBS reception at the laboratory in Hanoi, viral load was measured and then DBS cards were stored in a freezer at -20°C.

Prior to this study, in each of the 6 provinces, one training session was organized by the staff from the central laboratory. Two laboratory technicians per clinical site were invited to attend this training and were in charge of dissemination to their fellow laboratory co-workers at clinical site level. The training focused specifically on calibration of blood quantities to drop on each spot, maintenance before packing and DBS storage conditions and transfer. In some sites, technicians had prior experience with DBS for early infant diagnosis, however the rules for blood collection for HIV viral load monitoring are different and justified training them nevertheless. During on-site monitoring visits, laboratory staff were informally interviewed and reminded of good practice.

### Viral load measurement on DBS

HIV viral load was measured at the National Reference laboratory of HIV molecular biology at NIHE, Hanoi (Vietnam), using Abbott HIV-1 Real Time assay. Following the manufacturer’s recommendations, one single DBS spot of 70μL of whole blood was placed into a tube with 1.3 mL of Abbott mDBS buffer preparation (Abbott molecular, Des Plaines, IL). The tube was mixed by swirling and incubated for 30 minutes in a heating block (Benchmark Scientific) at 55°C before being directly loaded on the m2000sp platform for sample preparation. Amplification and detection was performed on the m2000rt platform using the open mode 1.0 mL HIV-1 RNA DBS IUO TT version 11 protocol. The lower limit of detection of the assay indicated by the manufacturer was 839 copies/mL.

### Statistical analysis

Baseline characteristics were summarized using frequencies and proportions for categorical variables, median and inter quartile range (IQR) for continuous variables; they were compared between PWID and other patients using chi-2 test for categorical variables and student t-test for continuous variables.

All viral load results, measured between 4 and 10 months after the date of ART initiation, were considered as 6 month evaluation. Virological failure was defined as having a viral load >1000 copies/mL at 6 months of ART. Baseline factors associated with virological failure were identified using logistic regression models. Factors associated with a p-value <0.20 in univariate analysis were considered in the multivariate model. Then factors independently associated with virological failure were identified through backward stepwise procedure.

All statistical analyses were performed using Stata 14 (Stata Corp., College Station, TX, USA). A p-value <0.05 was considered significant.

### Ethical considerations

The study protocol was approved by the Institutional Review Board from the National Institute of Hygiene and Epidemiology (Hanoi, Vietnam) (decision number 02/QD-VSDTTU), and the Institutional Review Board from Institut Pasteur (Paris, France) (decision number 2016-10/IRB/1). Authorization for data processing has been obtained from French legal authority (Commission Nationale Informatique et Liberté, decision number DR-2017-046). Only participants who provided written informed consent were enrolled, and a random anonymous identification number was assigned to each patient to guarantee confidentiality.

## Results

### Description of the population who initiated ART

A total of 578 patients were enrolled in the cohort and followed prospectively from the time of ART initiation.

Overall, 261 (45.1%) current or former PWID were enrolled (Table 1), of whom 97 (32.1%) were enrolled in a methadone-substitution program. Some care sites were within drug treatment centres and cared almost exclusively for PWID.

**Table 1:** Baseline characteristics between people who inject drug (PWID) and the other patients.

	Total (n = 578)	PWID (n = 261)	Other (n = 317)	p
Male gender	428 (74.0)	254 (97.3)	174 (54.9)	<0.001
Median (IQR) age at ART initiation (years)	33 (28–39)	35 (30–40)	32 (27–38)	0.002
Duration of known HIV infection (months)				
N (%)	571 (98.8)	258 (98.9)	313 (98.7)	
Median (IQR)	0.4 (0.2–0.8)	0.6 (0.2–1.1)	0.3 (0.1–0.7)	0.025
CD4 count at ART initiation (cells/mm^3^)^a^				
N (%)	162 (28.0)	62 (23.7)	100 (31.6)	0.038
Median (IQR)	287 (83–449)	277 (92–420)	291 (76–477)	0.63
WHO stage at ART initiation				0.46
1–2	456 (78.9)	199 (76.2)	257 (81.1)	
3	72 (12.5)	38 (14.6)	34 (10.7)	
4	23 (4.0)	12 (4.6)	11 (3.5)	
Missing	27 (4.7)	12 (4.6)	15 (4.7)	
BMI at ART initiation (kg/m^2^)				
N (%)	566 (97.8)	253 (96.9)	313 (98.4)	
Median (IQR)	19.8 (18.4–21.3)	19.8 (18.4–21.2)	19.8 (18.4–21.6)	0.31

PWID: people who inject drug; IQR: inter quartile range; BMI: body mass index

^a^ Measured in the interval -3 month / +15 days around the date of ART initiation

Most of the patients enrolled were male, the proportion of males was even higher in PWID. PWID were also significantly older at ART initiation, although only by a few years

After HIV diagnosis, the median delay to ART initiation was 0.3 months and was significantly longer in PWID (p = 0.034). The 1^st^ line ART combined lamivudine, tenofovir and efavirenz (3TC-TDF-EFV) in all patients. CD4 count was measured prior to ART initiation in only 162 (28%) patients, with this proportion being slightly lower in PWID (p = 0.040). When CD4 count was available, it did not differ between PWID and the other patients (p = 0.63); median CD4 level was 287 cells/mm^3^.

Overall, hepatitis B and C screening at the time of ART initiation was done in 49.7% and 47.1% of the patients, respectively. These proportions did not differ between PWID and other patients (p = 0.15 and p = 0.25, respectively). In those tested, 31 (10.8%) patients were HBV-infected, no difference was observed between PWID and the other patients (p = 0.98). On the other hand, significantly more patients were HCV-infected in PWID than in the other patients (91 (78.4%) and 14 (9.0%), respectively; p<0.001).

### Description of the system for virological monitoring using DBS

Of the 578 patients who initiated ART, 537 (92.9%) were still being followed 6 months after ART initiation. Of these patients, 397 (73.9%) had a DBS collected for the 6 month virological evaluation, 21 (3.9%) did not have a DBS collected because they did not attend a clinical visit in the 4–10 month time interval after the date of ART initiation, 115 (21.4%) did not have a DBS collected although they attended at least one clinical visit in the 4–10 month time interval (Table 2). Finally, in 4 patients the DBS was lost at some point thus virological evaluation could not be performed.

**Table 2:** Description of DBS network and virological evaluation at 6 month of ART.

Status at 6 month of ART (N = 578)	
Still followed	537 (92.9)
Dead	14 (2.4)
Transfer-out	15 (2.6)
Lost to follow-up	12 (2.1)
Patients still followed at 6 months of ART, with DBS	397 (73.9)
Patients still followed at 6 months of ART, without DBS	140 (26.1)
With visit between 4 and 10 months after the date of ART imitation	115
Without visit between 4 and 10 months after the date of ART imitation	21
DBS lost/never received	4
Timing of the DBS collection (in months)	
Median (IQR)	6.4 (5.8–7.2)
Delay from DBS collection to reception at the central laboratory (days)	
Median (IQR)	8 (6–19)
Delay from DBS reception at the central laboratory to viral load measurement (days)	
Median (IQR)	8 (7–15)
Overall delay from DBS collection to viral load measurement (days)	
Median (IQR)	22 (14–35)
≤30 days	278 (70.0)
Evaluation of the quality of the DBS at reception	
Desiccants showing sign of humidity at reception at the central laboratory	33 (8.3)
Desiccants showing sign of humidity at viral load measurement	-
<3 desiccants	3 (0.8)
<5 spots of whole blood on the card	2 (0.5)
Blood scattered outside of the circle	6 (1.5)
Frozen at intermediate transfer site	26 (6.5)
Viral load level (N = 373*)	
<1000 copies/mL	314 (84.2)
≥1000 copies/mL	59 (15.8)
Availability of confirmatory DBS (n = 59)	
Yes	15 (25.4)
No, death	1 (1.7)
No, lost to follow-up	3 (5.1)
No, no reason	40 (67.8)
Delay between the collection of the two samples (months) (n = 15)	
Median (IQR)	3.5 (3.2–4.6)
Confirmation of virological failure (n = 15)	
<1000 copies/mL	4 (25.6)
≥1000 copies/mL	11 (74.4)

* Results from 24 DBS were discarded because viral load was undetected but quality of DBS was not considered correct (i.e. desiccants showing signs of humidity or absence of desiccants)

DBS: dried blood spots; IQR: inter quartile range

After DBS collection at the HIV care site, the median (IQR) delay until reception at the central laboratory in Hanoi was 8 (6–19) days (Table 2). The median (IQR) delay from reception at the central laboratory to viral load measurement was 8 (7–15) days. Hence, the median (IQR) delay between DBS collection and viral load measurement was 22 (14–35) days, and was ≤30 days in 278 (70.0%) patients.

The quality of the DBS cards was evaluated upon reception at the central laboratory. Of the 397 samples received for the 6 month virological evaluation, 33 (8.3%) had desiccants showing signs of humidity and were then replaced; in 3 samples the number of desiccants did not fulfil recommendations (Table 2).

To try to understand who the patients without DBS collected for the 6 month virological evaluation were, we compared patients seen at M6 with DBS to those seen at M6 without DBS (S2 Table). The proportion of patients without DBS was significantly higher in the Thanh Hoa province compared to all other provinces, particularly in some clinical sites. Patients without DBS had lower CD4 count level at ART initiation (p = 0.04) and were more often not tested for HCV (p = 0.002). On the other hand, availability of DBS was not associated with gender, age, drug use, ethnicity, BMI level at ART initiation, WHO stage at ART initiation, remoteness to the clinical site or HBV status.

### Virological evaluation at 6 months of ART

Viral load was measured on all 397 DBS. However, out of the 33 DBS that showed signs of humidity at DBS reception at the central laboratory, 24 led to undetected viral load and were discarded as humidity could have impaired quantification, bringing into question the validity of these undetectable results. Hence, the virological results of 373 patients were considered. Of these, the viral load was ≥1000 copies/mL in 59 (15.8%) patients. Following national guidelines, these 59 patients should have had a second DBS collected to confirm virological failure, but this was done in only 15 (25.4%) of them, of whom 11 (73.3%) were confirmed in virological failure (Table 2).

Factors associated with initial failure (i.e. not considering the confirmatory measurement) were investigated (Table 3). In multivariate analysis, patients who had a low BMI at ART initiation and who self-reported not being fully adherent were more likely to be in virological failure (p = 0.011 and p<0.001, respectively). In some provinces, the risk of failure was increased. After adjusting for these factors, the distance to the care site was no longer associated with virological failure. Former or current drug use was not significantly associated with virological failure. HCV status was not known in about 50% of the patients, so this factor was not considered in the multivariate analysis.

**Table 3:** Factors associated with virological failure (1^st^ viral load ≥1000 copies/mL, N = 373).

	N	VL ≥1000 copies/mL (%)	Crude OR (95% CI)	p	Adj. OR (95% CI)	p
Province				<0.001		<0.001
Lai Chau	42	6 (14.3)	2.58 (0.68–9.77)		3.29 (0.86–12.51)	
Lao Cai	33	7 (21.2)	4.17 (1.12–15.48)		3.79 (1.01–14.26)	
Phu Tho	66	4 (6.1)	1		1	
Thai Nguyen	58	4 (6.9)	1.15 (0.27–4.81)		1.59 (0.38–6.60)	
Thanh Hoa	111	30 (27.0)	5.74 (1.92–17.15)		9.57 (3.04–30.14)	
Yen Bai	63	8 (12.7)	2.25 (0.64–7.90)		3.44 (0.96–12.38)	
Gender				0.07		
Male	268	48 (17.9)	1			
female	105	11 (10.5)	0.53 (0.27–1.08)			
Age (years)				0.75		
≤29	113	15 (13.3)	1			
30–39	163	27 (16.6)	1.30 (0.66–2.67)			
40–49	81	15 (18.5)	1.48 (0.68–3.24)			
≥50	16	2 (12.5)	0.93 (0.19–4.52)			
Drug use				0.49		
No	197	27 (13.7)	1			
Yes, not engaged in substitution prog.	123	22 (17.9)	1.37 (0.74–2.53)			
Yes, engaged in substitution prog.	53	10 (18.9)	1.46 (0.66–3.25)			
Distance to care site				0.012		
<10 km	93	11 (11.8)	1			
10 to 30 km	129	27 (20.9)	1.97 (0.92–4.21)			
>30 km	92	13 (14.1)	1.23 (0.52–2.90)			
Drug treatment centre	35	1 (2.9)	0.22 (0.02–1.77)			
unknown	24	7 (29.2)	3.07 (1.04–9.06)			
Time to care site						
<30 minutes	114	15 (13.2)	1	0.015		
30 to 60 minutes	118	25 (21.2)	1.77 (0.88–3.57)			
>60 minutes	82	11 (13.4)	1.02 (0.44–2.36)			
Drug treatment centre	35	1 (2.9)	0.20 (0.02–1.52)			
Unknown	24	7 (29.2)	2.72 (0.97–7.64)			
Provided supporter’s name				0.90		
No	46	7 (15.2)	1			
Yes	327	52 (15.9)	1.05 (0.45–2.48)			
Delay of ART initiation* (months)				0.52		
≤1	293	46 (15.7)	1			
1–12	58	8 (13.8)	0.86 (0.38–1.93)			
>12	19	5 (26.3)	1.79 (0.62–5.17)			
BMI at ART initiation (kg/m^2^)				0.034		0.012
<17	37	9 (24.3)	2.32 (1.02–5.28)		2.78 (1.14–6.78)	
17–18.5	65	16 (24.6)	2.32 (1.19–4.53)		2.85 (1.39–5.83)	
18.5–24.9	258	32 (12.4)	1		1	
>24.9	7	0	0.46 (0.03–8.33)		0.56 (0.08–11.28)	
Missing	6	2 (33.3)	3.87 (0.79–18.97)		4.41 (0.85–22.94)	
WHO stage at ART initiation				0.57		
1–2	304	45 (14.8)	1			
3	41	7 (17.1)	1.18 (0.50–2.84)			
4	14	4 (28.6)	2.30 (0.69–7.66)			
Missing	14	3 (21.4)	1.57 (0.42–5.85)			
Hepatitis B diagnosis^a^						
Not done	175	30 (17.1)	1.26 (0.71–2.24)	0.68		
Negative for HBs antigen	177	25 (14.1)	1			
Positive for HBs antigen	21	4 (19.0)	1.43 (0.44–4.60)			
Hepatitis C diagnosis^a^				0.039		
Not done	178	31 (17.4)	0.88 (0.48–1.62)			
Negative for anti-HCV antibodies	114	22 (19.3)	1			
Positive for anti-HCV antibodies	81	6 (7.4)	0.33 (0.13–0.87)			
Self-reported non-adherence				0.009		<0.001
No	326	45 (13.8)	1		1	
Yes	47	14 (29.8)	2.65 (1.32–5.33)		4.27 (1.90–9.59)	
Missed or delayed visits by >7 days				0.18		
No	291	42 (14.4)	1			
Yes	82	17 (20.7)	1.55 (0.83–2.90)			

VL: viral load; OR: odds ratio; CI: confidence interval; BMI: body mass index

* From the date of HIV confirmation

^a^ Measured in the interval -3 month / +1 month around the date of ART initiation

## Discussion

Despite the introduction of DBS use into the WHO guidelines and repeated arguments advocating for its use to widen access to HIV viral load monitoring in remote settings [15, 16], HIV viral load monitoring on DBS remains essentially limited to research settings where DBS results are compared to plasma results. Prior to this study, DBS use was reported in Democratic Republic of Congo, where HIV prevalence was low and patients widespread across the country, and where placement of new laboratories to measure viral load was not considered sustainable [17]. The non-governmental organization Médecins Sans Frontières (MSF; doctors without borders) also implemented viral load monitoring using DBS in some of its programmes, with variable viral load coverage [18]. Another study in Malawi evaluated the feasibility of DBS use for HIV viral load monitoring [19]. Our study is, therefore, one of the very few to use DBS routinely and to evaluate practical aspects of DBS use for routine HIV viral load monitoring in the field.

By simplifying transfer of blood samples and reducing its cost, DBS use is a pragmatic and immediately available option to offer routine virological monitoring to patients on ART in remote areas. We documented the various delays of viral load monitoring to see how DBS fitted into routine care. In Vietnam, clinical visits are planned monthly, or bimonthly when the patient is in a steady health state regarding HIV infection. The median delay between DBS collection at the clinical site and reception in Hanoi at the central laboratory was 8 days, showing the strong commitment of clinical teams. The overall delay between DBS collection and viral load measurement at the central laboratory was 22 days, and was ≤30 days in 70.0% of the patients. In the vast majority of patients, this delay was in line with the time interval between two consecutive clinical visits, meaning that the viral load results could be given back to the patient at the following the visit after DBS was collected. In some patients however, the delay was such that the result would be provided to the patient at the second clinical visit following DBS collection. Longer delays occurred when the clinical site did not transfer the samples weekly, or when viral load measurement was not performed in a timely manner at the central laboratory level. Logistical constraints or lack of familiarity with virological monitoring and DBS collection could explain these delays; these aspects must be addressed to offer better HIV care.

Worryingly, 4 DBS were declared to be lost after collection, with no ability to identify when, where and how this occurred. This study was conducted in real-life conditions, and although not anticipated, this was fortunately marginal. However, the clinical team should have been aware of this loss due to the lack of viral load result, but this was not the case, and no new DBS was collected. This illustrates that tracking of prescribed biological exams does not seem to exist. This has a direct impact at the individual level as these patients were not virologicaly evaluated. However, because these patients expected a viral load result that never came, this could lead to a mistrust in the health system and negatively affect compliance to clinical care and adherence to ART.

At reception at the central laboratory, some DBS exhibited defects that could have impaired virological evaluation. In particular, in 8.3% of the samples, desiccants showed signs of humidity. Rarely (n = 3, 0.8%), the number of desiccants did not comply with recommendations. Training dedicated to laboratory staff took place prior to the implementation of this study, concerning collection of DBS with calibrated blood quantities with single-use cones to prevent contamination as well as procedures for drying, packing, storage and transfer. The deficiencies observed could be due to lack of attention or a changing of staff and should be addressed by regular on-site training and awareness campaigns. Monitoring these quality indicators was important to prevent returning results that may falsely indicate virological control.

Another important issue to address is the absence of DBS collected in a significant proportion of patients at 6 months of ART. This was especially true in the Thanh Hoa province, and particularly so amongst some clinical sites. This situation occurred despite the fact that, prior to the implementation of the study, training were organised dedicated to clinical teams to define when DBS must be collected to comply with the national algorithm. This illustrates that one relies on the involvement and the good will of the clinical teams to manage routine virological monitoring. The impact on workload, at both the clinical and laboratory level, of the introduction of virological monitoring, in sites that did not previously have access to it, should not be minimized and could explain the lack of DBS collection. A recent review emphasized the need and the importance to train clinicians on how to incorporate viral load monitoring in their practice [15]. MSF also insists on the importance of continuous training as to viral load monitoring [18]. A study in Malawi revealed that the introduction of routine viral load monitoring could be felt as a new burden in the case of a shortage of staff [20]. During the course of the study, staff departure or transfer occurred, which could have resulted in increased workload and the arrival of untrained staff which may have induced the flaws apparent in the routine virological monitoring. This also calls for regular campaigns and training to ingrain clinical staff as to the importance of routine viral load monitoring.

This DBS evaluation study was implemented shortly after Vietnam implemented a test-and-treat strategy in order to increase the number of patients on ART. In 2018, it was estimated that 65% of those who are HIV-infected are on ART [1]. In those newly initiating ART, nearly 50% were PWID, formerly or currently using drugs. This result follows the elevated HIV incidence in PWID in Vietnam [1] and is in line with previous findings [21, 22]. Patients newly initiating ART were essentially men, a finding also highlighted in other studies in Vietnam [6, 23–26]. This is partly explained by the large representation of PWID in this study; nearly all PWID were male, as previously reported [27]. In Vietnam, the HIV epidemic is also concentrated in MSM and female sex worker (FSW). However, few men identified as MSM and only 3 patients identified as FSW in this study. The under-representation of women in HIV programmes in Vietnam may result from the HIV-related communication being essentially targeted towards key-populations; it is possible that the general population does not feel at risk and hence does not get tested, especially women [28].

As a probable consequence of the test-and-treat strategy, CD4 measurements were no longer measured systematically prior to ART initiation, indeed only 28% of the patients had a CD4 measurement. In this group, the median (IQR) CD4 level was 287 (83–449) cells/mm^3^, which is higher than in previous reports in Vietnam [24, 29] and in line with the global increase of CD4 level at ART initiation observed in resource-limited settings [30].

In terms of virological results, the proportion of patients with a viral load <1000 copies/mL at 6 months was 84.2%. This is lower than other evaluations which took place at later time points [23, 24, 31], but similar to the proportion observed in a test-and-treat evaluation study in PWID performed in Vietnam [29]. At later time points, the higher rate of virological success could be explained by a survival bias effect as those who were not fully adherent are no longer in active care.

A lower BMI at ART initiation was significantly associated with increased risk of failure. Lower BMI has been found to be associated with mortality [26, 32, 33]. Reasons behind low BMI should be addressed to improve BMI status and thus ART efficacy. Interestingly, those self-identifying as not fully adherent were more likely to be in virological failure, suggesting that targeting patients who need strengthened support for adherence could prove useful. Finally, the rate of virological success differed significantly according to the province. This reflects disparities between clinical sites that were not captured by the factors at our disposal.

Although the national guidelines are, in case of a viral load ≥1000 copies/mL, to provide adherence support and additional viral load testing within 3 to 4 months, this was not achieved in all patients. Only 14 (25.8%) out of 58 patients with primary failure had a confirmatory viral load. Other studies have reported low proportions of patients receiving confirmatory viral load on time, with even worse results [34, 35]. Prior to this study, care sites did not have access to routine viral load monitoring and training was organized to explain the algorithm. It is possible that health staffs working at OPC did not sufficiently understand the need and conditions for confirmatory viral load. It has also been reported that the attendance of all visits necessitated by viral load monitoring was challenging for patients [20]. All this could have contributed to the lack of confirmatory viral load. However, a better understanding of the reasons behind this lack of confirmatory viral load is crucial for the program to work efficiently and to be able to be addressed.

Of those with confirmatory viral load, the majority were confirmed as failures. This is a lot more than usually observed [36], but could be explained by a selection bias from the clinical staff.

Our study has some limitations, the main one being that it is a descriptive study in real-life conditions. Apart from training, no intervention was implemented at the clinician or laboratory levels, thus explaining the delays and DBS coverage. We reported a slightly lower rate of virological success than previous studies [23, 24, 31], and one could object that DBS use could explain this due to the proviral DNA amplification [37]. However, the technique used for viral load measurement on DBS, which is CE-marked, performed well in an evaluation in the same laboratory [14]. Of note, another evaluation in PWID in Vietnam reported a very similar virological failure rate [29].

This study was the first one to use DBS for routine HIV viral load monitoring in the country. The results and progresses from this study are regularly discussed with the Vietnam Administration of HIV/AIDS control (VAAC) that is part of the Ministry of Health. These results have informed the decision from the Ministry of Health to allow DBS use for HIV viral load monitoring in March 2019. Discussions are still on-going on the scaling-up of DBS use in remote settings. In a first stage, DBS use could be opened to all HIV patients followed in the clinical sites that participated in the study, and in a second stage progressively be opened to all the clinical sites caring for HIV patients in these provinces and eventually in other remote provinces where DBS could help implement routine viral load monitoring.

## Conclusion

This study documents that DBS use for routine viral load monitoring is feasible in remote areas and that its use fits well within the framework of HIV care. Suboptimal coverage for viral load monitoring and for confirmation of virological failure illustrates the need of adapted training to ensure the strong involvement of clinical teams.

## Supporting information

S1 Table Baseline characteristics of the patients. (DOCX)

S2 Table Comparison of patients with and without DBS at 6 months. (DOCX)
