# Supplementary material for: Feasibility of dried blood spots for HIV viral load monitoring in decentralized area in North Vietnam in a test-and-treat era, the MOVIDA project
Source: PLoS One. 2020 Apr 9;15(4):e0230968. doi: 10.1371/journal.pone.0230968 (PMC7145146; doi:10.1371/journal.pone.0230968)
Supplement: S1 Table — (DOCX) [file pone.0230968.s001.docx]

Supplementary table 1: Baseline characteristics of the patients

|  | Total  (n=578) | PWID  (n=261) | Other  (n=317) | p |
| --- | --- | --- | --- | --- |
| Province  Lai Chau  Lao Cai  Phu Tho  Thai Nguyen  Thanh Hoa  Yen Bai | 53 (9.2)  51 (8.8)  77 (13.3)  71 (12.3)  242 (41.9)  84 (14.5) | 26 (10.0)  29 (11.1)  39 (14.9)  24 (9.2)  93 (35.6)  50 (19.2) | 27 (8.5)  22 (6.9)  38 (12.0)  47 (14.8)  149 (47.0)  34 (10.8) | 0.002 |
| Ethnicity  Kinh  Other  Not specified | 348 (60.2)  201 (34.8)  29 (5.0) | 151 (57.9)  93 (35.6)  17 (6.5) | 197 (62.1)  108 (34.1)  12 (3.8) | 0.26 |
| Marital status  Single  Married  Divorced/widowed | 147 (25.4)  348 (60.2)  83 (14.4) | 79 (30.3)  151 (57.8)  31 (11.9) | 68 (21.5)  197 (62.1)  52 (16.4) | 0.032 |
| Sedentary occupation  No  Yes  Not specified | 120 (20.8)  411 (71.1)  47 (8.1) | 39 (14.9)  187 (71.7)  35 (13.4) | 81 (25.5)  224 (70.7)  12 (3.8) | <0.001 |
| Housing equipped with running water  No  Yes  Not specified | 167 (28.9)  403 (69.7)  8 (1.4) | 72 (27.6)  185 (70.9)  4 (1.5) | 95 (30.0)  218 (68.8)  4 (1.3) | 0.80 |
| Provided treatment supporter’s name | 504 (87.2) | 207 (79.3) | 297 (93.7) | <0.001 |
| Provided contact details | 560 (96.9) | 249 (95.4) | 311 (98.1) | 0.06 |
| Hepatitis B diagnosis^a^  Not done  Negative for HBs antigen  Positive for HBs antigen | 291 (50.3)  256 (44.3)  31 (5.4) | 140 (53.6)  108 (41.4)  13 (5.0) | 151 (47.6)  148 (46.7)  18 (5.7) |  |
| Hepatitis C diagnosis^a^  Not done  Negative for anti-HCV antibodies  Positive for anti-HCV antibodies | 306 (52.9)  167 (28.9)  105 (18.2) | 145 (55.6)  25 (9.6)  91 (34.9) | 161 (50.8)  142 (44.8)  14 (4.4) |  |

PWID: people who inject drug; IQR: inter quartile range; BMI: body mass index

^a^ Measured in the interval -3 month / +1 month around the date of ART initiation
