# Supplementary material for: Feasibility of dried blood spots for HIV viral load monitoring in decentralized area in North Vietnam in a test-and-treat era, the MOVIDA project
Source: PLoS One. 2020 Apr 9;15(4):e0230968. doi: 10.1371/journal.pone.0230968 (PMC7145146; doi:10.1371/journal.pone.0230968)
Supplement: S2 Table — (DOCX) [file pone.0230968.s002.docx]

Supplementary Table 2: Comparison of patients with and without DBS at 6 months

|  | With DBS  (n=397) | Without DBS  (n=115) | P |
| --- | --- | --- | --- |
| Province  Lai Chau  Lao Cai  Phu Tho  Thai Nguyen  Thanh Hoa  Yen Bai | 42 (10.6)  33 (8.3)  67 (16.9)  58 (14.6)  131 (33.0)  66 (16.6) | 7 (6.1)  7 (6.1)  4 (3.5)  6 (5.2)  78 (68.7)  12 (10.4) | <0.001 |
| Ethnicity  Kinh  Other  Not specified | 239 (60.2)  138 (34.8)  20 (5.0) | 65 (56.5)  44 (38.3)  6 (5.2) | 0.77 |
| Male gender | 287 (72.3) | 89 (77.4) | 0.28 |
| Median (IQR) age at ART initiation (years) | 33 (28-40) | 33 (28-38) | 0.39 |
| Drug use  No  Yes, not enrolled in substitution program  Yes, enrolled in substitution program | 215 (54.2)  125 (31.4)  57 (14.4) | 68 (59.1)  26 (22.6)  21 (18.3) | 0.16 |
| BMI at ART initiation (kg/m²)  N (%)  Median (IQR) | 389 (98.0)  20.0 (18.4-21.4) | 114 (99.1)  19.8 (18.4-21.4) | 0.91 |
| Housing equipped with running water  No  Yes  Not specified | 119 (30.0)  272 (68.5)  6 (1.5) | 32 (27.8)  82 (71.3)  1 (0.9) | 0.90 |
| CD4 count at ART initiation (cells/mm^3^)^a^  N (%)  Median (IQR) | 119 (30.0)  321 (97-458) | 22 (19.1)  133 (25-378) | 0.02  0.04 |
| WHO stage at ART initiation  1-2  3  4  Missing | 321 (80.9)  42 (10.6)  14 (3.5)  20 (5.0) | 95 (82.6)  12 (10.4)  4 (3.5)  4 (3.5) | 0.96 |
| Distance to care site  <10 km  10 to 30 km  >30 km  Drug treatment centre  unknown | 97 (24.4)  138 (34.8)  98 (24.7)  36 (9.1)  28 (7.0) | 36 (31.3)  45 (39.1)  26 (22.6)  6 (5.3)  2 (1.7) | 0.09 |
| Time to care site  <30 minutes  30 to 60 minutes  >60 minutes  Drug treatment centre  Unknown | 118 (29.7)  128 (32.2)  87 (21.9)  36 (9.1)  28 (7.0) | 40 (34.7)  38 (33.0)  28 (24.3)  6 (5.3)  2 (1.7) | 0.24 |
| Hepatitis B diagnosis^b^  Not done  Negative for HBs antigen  Positive for HBs antigen | 188 (47.4)  188 (47.4)  21 (5.2) | 65 (56.5)  42 (36.5)  8 (7.0) | 0.12 |
| Hepatitis C diagnosis^b^  Not done  Negative for anti-HCV antibodies  Positive for anti-HCV antibodies | 191 (48.1)  121 (31.5)  81 (20.4) | 76 (66.1)  28 (24.3)  11 (9.6) | 0.002 |

DBS: dried blood spots; IQR: inter quartile range: PWID: people who inject drug; BMI: body mass index; WHO: world health organization

^a^ Measured in the interval -3 month / +15 days around the date of ART initiation

^b^ Measured in the interval -3 month / +1 month around the date of ART initiation
